# Effects of physical exercise on metabolic syndrome in psychotic disorders: A systematic review with meta-analysis of randomized controlled trials

**DOI:** 10.1192/j.eurpsy.2025.10064

**Published:** 2025-07-18

**Authors:** Arantxa Ancín-Osés, Mikel Izquierdo, Manuel J. Cuesta, Mikel L. Sáez de Asteasu

**Affiliations:** 1 Navarrabiomed, Hospital Universitario de Navarra (HUN), Universidad Pública de Navarra (UPNA), IdiSNA, Pamplona, Spain; 2 CIBER of Frailty and Healthy Aging (CIBERFES), Instituto de Salud Carlos III, Madrid, Spain; 3 Department of Psychiatry, Hospital Universitario de Navarra, Pamplona, Spain; 4 Instituto de Investigación Sanitaria de Navarra (IdiSNA), Pamplona, Spain

**Keywords:** metabolic syndrome, physical exercise, psychotic disorder, schizophrenia, severe mental disorder

## Abstract

**Background:**

Physical exercise improves mental and physical health of individuals with severe mental illness (SMI); however, its impact on metabolic syndrome remains unclear.

**Aims:**

To evaluate the effects of exercise interventions on metabolic syndrome components in individuals with SMI and explore interactions between exercise and antipsychotic medications on metabolic outcomes.

**Methods:**

Following PRISMA guidelines, we systematically searched PubMed, CINAHL, Web of Science, and APA PsycINFO through October 10, 2023, for randomized controlled trials (RCTs) assessing the effects of exercise on waist circumference, blood pressure, glucose, triglycerides, and HDL cholesterol in SMI. Risk of bias was evaluated using the Cochrane RoB-2 tool. Data were pooled using random-effects models in Comprehensive Meta-Analysis and JASP.

**Results:**

Ten RCTs (*N* = 773; mean age 39.9 ± 7.36 years; 38.7% female; 71.5% schizophrenia spectrum disorders) met inclusion criteria. Pooled analyses revealed no significant effects of exercise on waist circumference (SMD = 0.206, 95% CI [−0.118, 0.530], *p* = 0.171), systolic blood pressure (SMD = 0.194, 95% CI [−0.115, 0.504], *p* = 0.219), diastolic blood pressure (SMD = −0.21, 95% CI [−0.854, 0.434], *p* = 0.522), HDL (SMD = 0.157, 95% CI [−0.36, 0.674], *p* = 0.551), triglycerides (SMD = −0.041, 95% CI [−0.461, 0.38], *p* = 0.849), or glucose (SMD = −0.071, 95% CI [−0.213, 0.071], *p* = 0.326). Heterogeneity was moderate to high.

**Conclusions:**

Exercise interventions did not significantly improve metabolic syndrome components in SMI. Future trials must prioritize tailored regimens, adjunctive therapies, and rigorous control of medication effects.

## Introduction

Severe mental disorders (SMI) characterized by extreme disturbances in cognition, emotional regulation, and behavior create significant burdens on psychosocial and occupational functioning. It is often exacerbated by neurobiological and genetic factors that shape its course and treatment response [1, 2]. These conditions lead to a mortality gap of 10–20 years compared to the general population, in part, contributing to premature cardiovascular disease (CVD), which accounts for 14.3% of global annual deaths [3–6]. Emerging evidence underscores metabolic syndrome, a cluster of central obesity, hypertension, dyslipidemia, and hyperglycemia, as critical mediators of CVD risk in this population [7, 8].

The pathophysiology of SMI-related metabolic syndrome is multifactorial and involves chronic inflammation, immune dysregulation, and genetic susceptibility [9–11]. Second-generation antipsychotics worsen these risks owing to weight gain, insulin resistance, and lipid abnormalities [12]. At the same time, both antidepressants and mood stabilizers have milder metabolic effects, and polypharmacy and high-dose regimens lead to further compound adverse outcomes [13–16]. Schizophrenia, affecting 1% of the global population, underlies these challenges: prolonged antipsychotic treatment and chronicity of the disease combined to increase metabolic morbidity [17–20].

Nonpharmacological interventions, primarily structured exercises, have great potential to reduce risks [21]. Meta-analyses have shown that physical activity improves psychiatric symptoms, functional capacity, and quality of life in SMI [22–26]. Exercise also enhances cardiorespiratory fitness and reduces obesity, diabetes, and CVD incidence, which is vital for a population with twice the occurrence of metabolic syndrome compared to the general population [22, 27–29]. However, there are still some important gaps. First, exercise does not directly ameliorate the components of metabolic syndrome (e.g., waist circumference and blood pressure) in patients with psychotic disorders. Second, potential synergies or antagonisms between exercise and antipsychotic pharmacodynamics have not been examined despite evidence that physical activity may modulate drug metabolism and receptor sensitivity [30–32]. Such multidimensional advancements enhance global health and address the leading health-related issues in this group, thereby minimizing the likelihood of severe long-term repercussions [33, 34].

This systematic review and meta-analysis aimed to (1) evaluate the efficacy of physical exercise on metabolic syndrome components in psychotic disorders and (2) explore the interactions between exercise and antipsychotic medications on metabolic outcomes. By elucidating these mechanisms, our findings aim to inform integrative treatment paradigms that optimize the mental and physical health.

## Methods

### Study design

This systematic review and meta-analysis followed the guidelines outlined in the Preferred Reporting Items for Systematic Reviews and Meta-Analyses (PRISMA) statement [35] and was registered in PROSPERO (CRD42025635273).

### Search strategy

The search for studies was conducted on October 10, 2024. Articles were gathered from the Medline (PubMed), CINAHL, Web of Science, and APA PsycINFO databases. Two researchers independently screened the articles by evaluating their titles and abstracts to assess the eligibility criteria, and disagreements were resolved through discussion with a third author. The articles were screened using the Sysrev software. The search used a defined set of keywords related to physical exercise, metabolic syndrome, schizophrenia, psychotic disorders, and randomized controlled trials. The complete search strategy is available in the Supplementary Materials.

### Selection criteria

The PICOS model defined the inclusion criteria (Population, Intervention, Comparison, Outcome, Study type) [36]. The inclusion of studies was based on the following criteria: (a) studies focused mainly on people over 18 years with a diagnosis of psychotic disorder based on the *Diagnostic and Statistical Manual of Mental Disorders* (*DSM-V*) [1]; (b) randomized controlled trial (RCT); (c) assessed components of metabolic syndrome including waist circumference, blood pressure, fasting glucose, triglycerides, and HDL cholesterol; and (d) evaluation of exercise intervention. Systematic reviews, meta-analyses, single-group trials, practice guidelines, recommendations, editorials, letters, conference abstracts, reports, protocols, and studies with undefined interventions or procedures were excluded. We also excluded RCTs based on other specific SMI (i.e., bipolar disorder, anxiety, and major depressive disorder).

While schizophrenia was the primary diagnosis, we included related *DSM-V*-defined psychotic disorders (e.g., schizoaffective disorder and schizophreniform disorder). These conditions share core clinical and neurobiological features with schizophrenia, including antipsychotic treatment patterns and an elevated cardiometabolic risk. This broader “psychotic disorders” grouping enabled a more comprehensive evidence synthesis and increased statistical power.

### Screening

The search strategy gathered the results from four databases and eliminated duplicates. The titles and abstracts of the studies were screened according to eligibility criteria. Full-text articles were then evaluated, and those that met the eligibility criteria were selected for inclusion.

### Data extraction

Two authors independently extracted summary data based on key observations. They used a standardized form to collect relevant information, such as authors, publication year, study population, design, sample size, interventions, and outcomes. The third author resolved any disagreements during the selection or extraction process.

### Risk of bias assessment

Two authors independently evaluated the risk of bias for each included trial using the Cochrane Risk of Bias 2 (RoB-2) tool [37]. This tool examines domains such as the randomization process, adherence to interventions, handling of missing outcome data, measurement of the outcome, and selection of reported results. Each domain and trial was adjudicated based on a low-, some concerns, or high-risk level. Any disagreement between the two assessors was resolved through discussion with a third investigator. The overall quality of evidence was assessed using the Grading of Recommendations, Assessment, Development, and Evaluation (GRADE) approach.

### Statistical analysis and data synthesis

Statistical analysis for this meta-analysis was conducted using the Comprehensive Meta-Analysis and JASP statistical software [38, 39]. Effect sizes were computed using standardized mean differences (SMD) and 95% confidence intervals (CI) to measure the impact of physical exercise on the components of metabolic syndrome. A random-effects model was used to account for potential heterogeneity between the studies [40]. Heterogeneity was assessed using the *I*^2^ statistic, with thresholds of 25, 50, and 75% representing low, moderate, and high heterogeneity, respectively [41]. Additionally, *Q* statistics were used to determine statistical significance. Data synthesis focused on pooled estimates for each metabolic component. Publication bias was evaluated using selection models and Egger’s test. The significance level was set at *p* < 0.05.

## Results

### Study selection

**Figure 1.**
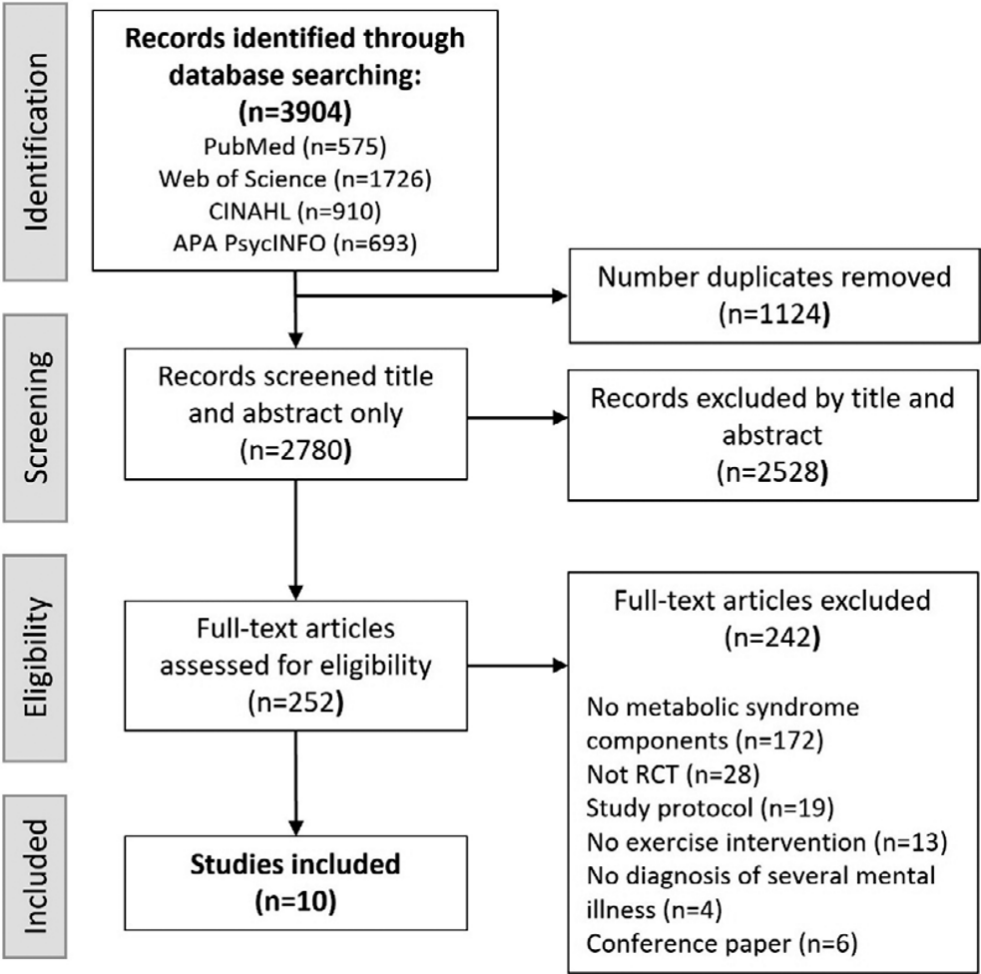
PRISMA flowchart of the study.

Figure 1 shows the flow diagram of the database search process. A total of 3904 articles were retrieved from these four databases. After removing 1124 duplicate articles, 2780 were screened by title and abstract, and 2528 were excluded. Finally, 252 articles remained for full-text screening, and 242 were excluded based on the inclusion and exclusion criteria. Finally, 10 articles were included in the review [42–51].

### Risk of bias assessment

**Figure 2.**
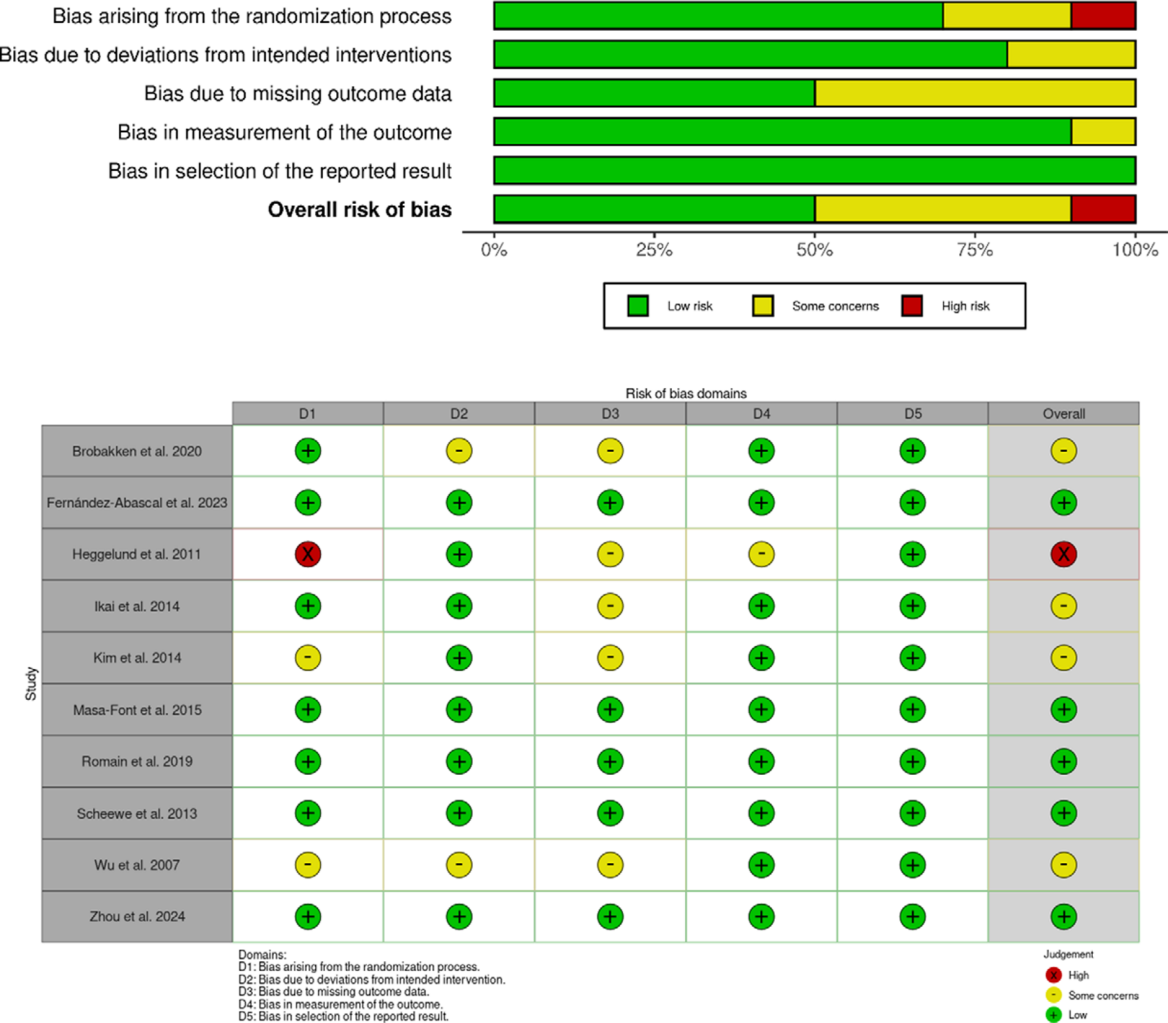
Risk of bias assessment. (A) Quality of methodology in the included studies. (B) Distribution of methodological quality across included studies.

Figure 2 shows the risk of bias across included studies. Five studies were classified as having a low risk of bias, four showed some concerns, and one was rated as having a high risk of bias. The primary source of bias was the blinding of the participants and personnel. However, randomization is mentioned in the studies; the lack of detailed information regarding the allocation process and whether concealment was properly implemented raise concerns about the procedure’s validity. Furthermore, some studies have reported challenges in participant adherence to interventions. However, the methods employed to manage these cases were not sufficiently detailed in the analysis, raising concerns regarding their potential impact on the overall risk of bias.

### Characteristics of the participants

Ten RCTs were included in this meta-analysis. The characteristics of the included RCTs are shown in Table 1. Two studies were performed in Spain [48, 51], two in Norway [43, 49], and one in Japan [45], the Netherlands [46], Canada [44], Taiwan [50], China [42], and the Republic of Korea [47]. A total of 773 participants were randomized into exercise (405 participants) and control groups (368 participants). In all, 71.54% had schizophrenia; 13.86% had schizoaffective disorder; and 2.12%, 1.25%, and 11.23% had other SMI, schizophreniform disorders, psychotic disorders, and other mental disorders (*DSM-V*) [1]. The mean age of the patients was 39.9 years (standard deviation [SD]: 7.36). Of the 10 RCTs included, 9 reported the participants’ sex [42–46, 48–51], of which 38.7% were female.

**Table 1.** Qualitative analysis of included studies analyzing the effects of exercise on the components of metabolic syndrome

References	Participants characteristics (*N*, % women, mean [SD] age, years)	Intervention characteristics	Evaluated components of metabolic syndrome	Results (between-group differences)
Wu et al. [50]	53 (IG = 28, CG = 25), 58.49% women, mean age: 40.6 (5.03)	Aerobic exercise, 60 min/session, 3*/week, for 6 months	Waist circumference, glucose, triglycerides	↓ Waist circumference (*p* < 0.001) = Glucose (*p* = 0.326) ↓ Triglycerides (*p* = 0.049)
Heggelund et al. [43]	19 (IG = 12, CG = 7), 31.58% women, mean age: 34.7 (10.05)	High-intensity aerobic training, 45 min/session, 3*/week, for 8 weeks, intensity: 85–95% HR_peak_/rest 70% HR_peak_	Blood pressure, glucose, triglycerides, HDL cholesterol	= Systolic blood pressure (*p* = 0.5) = Diastolic blood pressure (*p* = 0.4) = Glucose (*p* = 0.6) = Triglycerides (*p* = 0.4) ↑ HDL cholesterol (*p* = 0.007)
Scheewe et al. [46]	63 (IG = 31, CG = 32), 26.98% women, mean age: 29.65 (7.45)	Concurrent training (aerobic and resistance training), 60 min/session, 2*/week, for 6 months, intensity: 45–75% HRR	Waist circumference, blood pressure, glucose, triglycerides, HDL cholesterol	= Waist circumference (*p* = 0.591) = Systolic blood pressure (*p* = 0.249) = Diastolic blood pressure (*p* = 0.242) = Glucose (*p* = 0.721) = Triglycerides (*p* = 0.19) = HDL cholesterol (*p* = 0.115)
Ikai et al. [45]	50 (IG = 25, CG = 25), 34% women, mean age: 50.9 (11.3)	Hatha yoga, 60 min/session, 1*/week, for 8 weeks	Glucose, triglycerides, HDL cholesterol	= Glucose (*p* = 0.324) = Triglycerides (*p* = 0.240) = HDL cholesterol (*p* = 0.829)
Jae et al. [47]	36 (IG = 24, CG = 12), biological sex was not reported, mean age: 49.7 (10.09)	Concurrent training (aerobic and resistance training), 60 min/session, 2*/week, for 12 weeks, intensity: 50–70% HRR	Waist circumference, blood pressure, glucose, HDL cholesterol	↓ Waist circumference (*p* = 0.032) ↓ Systolic blood pressure (*p* = 0.018) ↓ Diastolic blood pressure (*p* = 0.024) = Glucose (*p* = 0.912) = HDL cholesterol (*p* = 0.331)
Masa-Font et al. [51]	332 (IG = 169, CG = 163), 45.2% women, mean age: 46.7 (6.6)	Structured walking sessions, 40–60 min/session, 2*/week, for 12 weeks	Waist circumference, blood pressure, glucose, triglycerides	= Waist circumference (*p* = 0.133) = Systolic blood pressure (*p* = 0.964) = Diastolic blood pressure (*p* = 0.407) ↑ Glucose (*p* = 0.035) = Triglycerides (*p* = 0.415)
Romain et al. [44]	66 (IG = 38, CG = 28), 37.88% women, mean age: 30.73 (7.23)	HIIT, 30 min/session, 2*/week, for 6 months. intensity: sprint 80–90% HR_max_/rest 50–65% HR_max_	Waist circumference, blood pressure, glucose, triglycerides, HDL cholesterol	= Waist circumference (*p* = 0.25) = Systolic blood pressure (*p* = 0.98) = Diastolic blood pressure (*p* = 0.18) = Glucose (*p* = 0.72) = Triglycerides (*p* = 0.76) = HDL cholesterol (*p* = 0.09)
Brobakken et al. [49]	48 (IG = 25, CG = 23), 41.66% women, mean age: 35 (1.75)	Aerobic interval training, 35 min/session, 2*/week, for 1 year, intensity: 70–95% HR_peak_	Waist circumference, blood pressure, glucose, triglycerides, HDL cholesterol	= Waist circumference (*p* = 0.79) = Systolic blood pressure (*p* = 0.93) = Diastolic blood pressure (*p* = 0.20) = Glucose (*p* = 0.72) = Triglycerides (*p* = 0.73) = HDL cholesterol (*p* = 0.81)
Fernández-Abascal et al. [48]	48 (IG = 24, CG = 24), 39.58% women, mean age: 44.72 (9.7)	Aerobic training, 120 min/session, 3*/week, for 12 weeks, intensity: progressive to 75% HR_max_	Waist circumference, blood pressure, glucose, triglycerides, HDL cholesterol	↓ Waist circumference (*p* = 0.045) = Systolic blood pressure (*p* = 0.699) = Diastolic blood pressure (*p* = 0.110) = Glucose (*p* = 0.197) = Triglycerides (*p* = 0.349) = HDL cholesterol (*p* = 0.059)
Zhou et al. [42]	58 (IG = 29, CG = 29), 0% women, mean age: 55.4 (3.8)	Dance, 60 min/session, 2 */week, for 12 weeks	Glucose, triglycerides	= Glucose (*p* = 0.13) = Triglycerides (*p* = 0.47)

### Interventions’ characteristics

Wide heterogeneity was observed during the intervention period. Exercise interventions lasted between 8 weeks and 12 months. Twelve weeks was the most frequently observed duration [42, 47, 48, 51], and two studies reported follow-up periods of 8 weeks and 18 months postexercise [45, 48]. Physical exercise programs involved one to three sessions per week, with each session lasting between 30 and 120 min.

Most of the intervention groups focused on aerobic exercise, including high-intensity interval training (HIIT) (*n* = 2) [43, 44], aerobic interval training (*n* = 1) [49], aerobic exercise including fast walking and stair climbing (*n* = 2) [48, 50], Hatha yoga (*n* = 1) [45], dance movements (*n* = 1) [42], and structured walking sessions (*n* = 1) [51]. Additionally, many studies combined aerobic and resistance training (*n* = 2) [46, 47].

Most studies provided usual medical care treatment, with ongoing psychiatric follow-ups (*n* = 6) [42, 44, 45, 48, 50, 51]. In contrast, Brobakken et al. [49] performed two supervised aerobic interval training (AIT) sessions. The participants were instructed to practice the exercises at home (*n* = 1) [49]. Other studies included occupational therapy sessions (*n* = 1) [46], weekly recreational sessions (*n* = 1) [47], and computer-based training sessions (*n* = 1) [43] for control participants.

The intensity of exercise interventions varied across the included studies. Two studies prescribed combined aerobic and resistance exercises at 45–75% of the heart rate reserve (HRR) [46, 47]. Other studies included HIIT sessions conducted at intensities ranging from 50% to 90% HR_max_ [44] and 70% to 95% HR_peak_ [43, 44], whereas AIT sessions were undertaken in a range of 70% to 75% HR_peak_ [49]. Another study included aerobic exercise sessions with increasing intensity up to 75% maximum HR [48]. Many studies did not prescribe exercise considering the intensity due to the nature of the interventions [42, 45, 50, 51]. These intensity variations were tailored to the specific demands of each intervention.

## Components of metabolic syndrome

### Waist circumference

**Figure 3.**
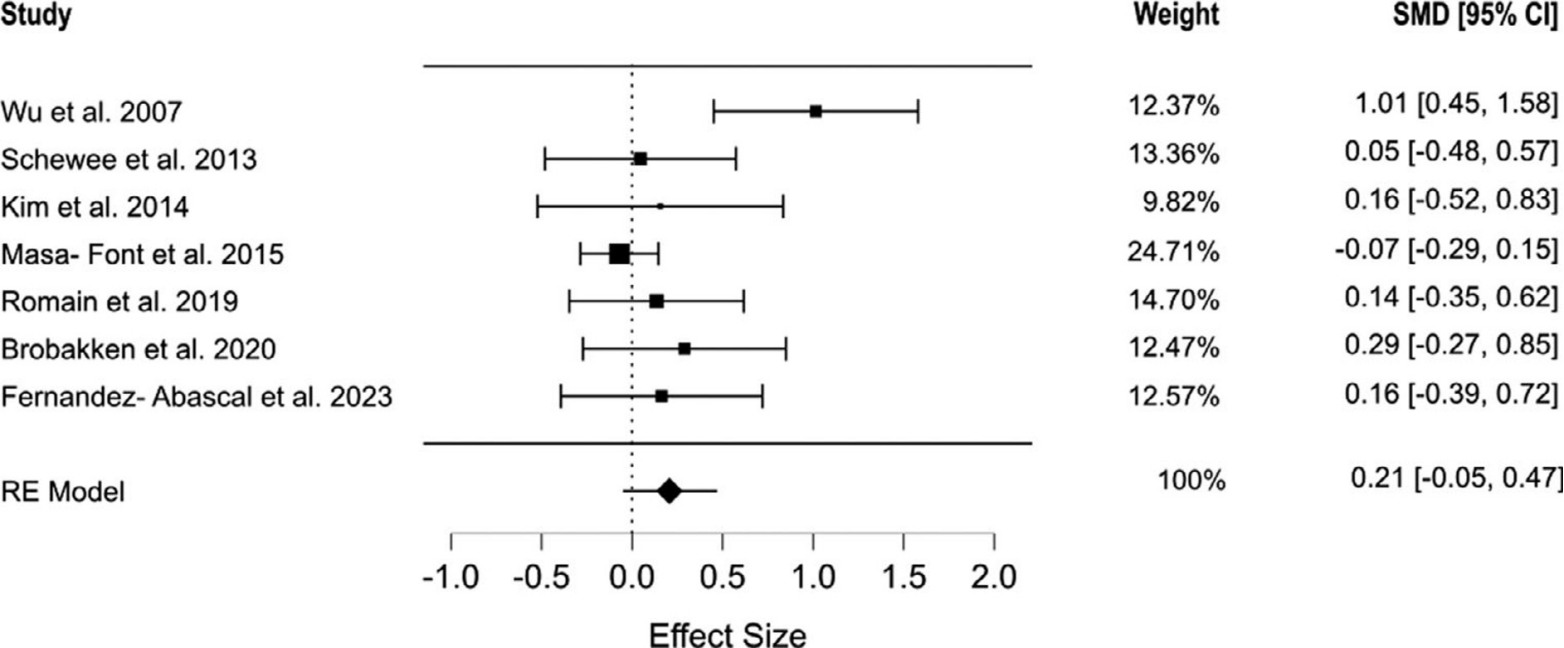
Forest plot of the effect of physical exercise on waist circumference.

Seven studies assessed the waist circumference [44, 46–51]. The pooled results indicated that exercise interventions did not significantly affect waist circumference (SMD = 0.206, 95% CI [−0.118, 0.530], *p* = 0.171). Moderate heterogeneity was observed among the included studies (*Q* = 13.054, *I*^2^ = 51.6%, *p* = 0.042) (Figure 3). The selection models reported no evidence of publication bias (*p* = 0.07), which was in line with the results of Egger’s regression (*p* = 0.18). The mean model estimates are presented in Supplementary Figure S1.

### Blood pressure

**Figure 4.**
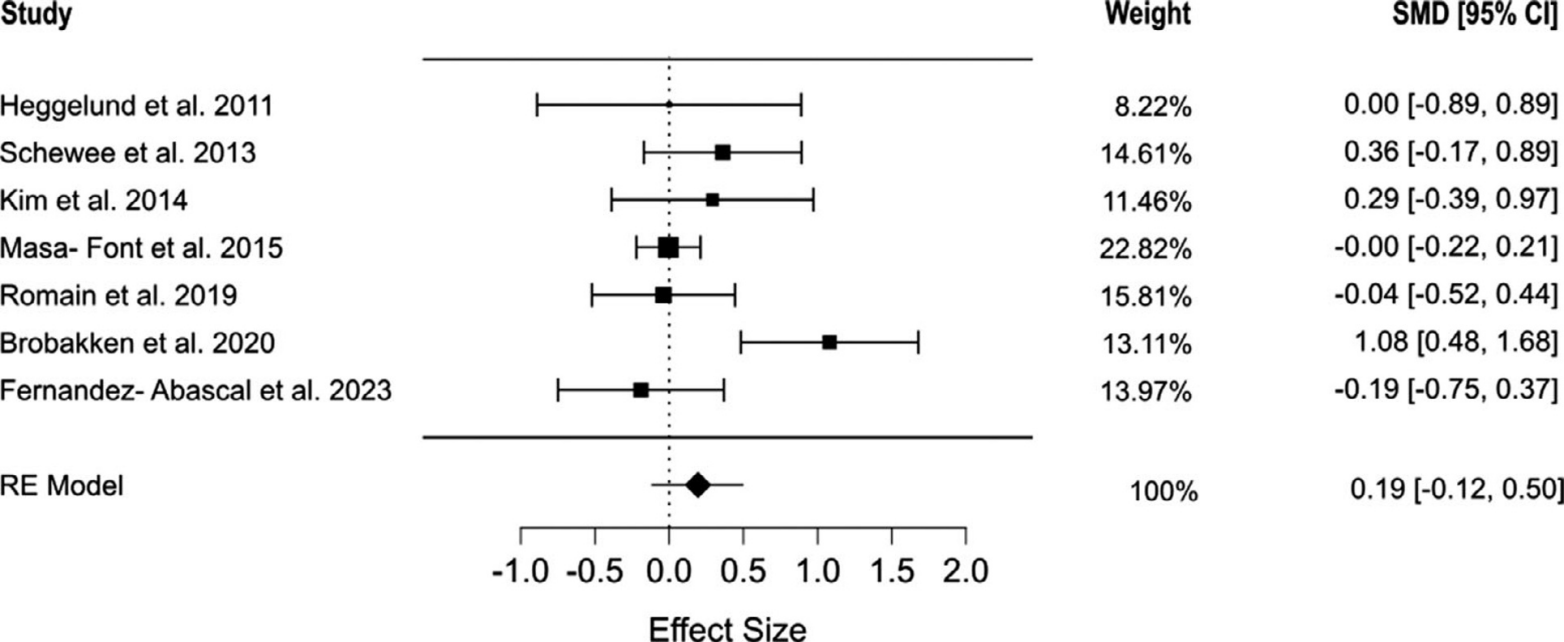
Forest plot showing the effect of physical exercise on systolic blood pressure.

**Figure 5.**
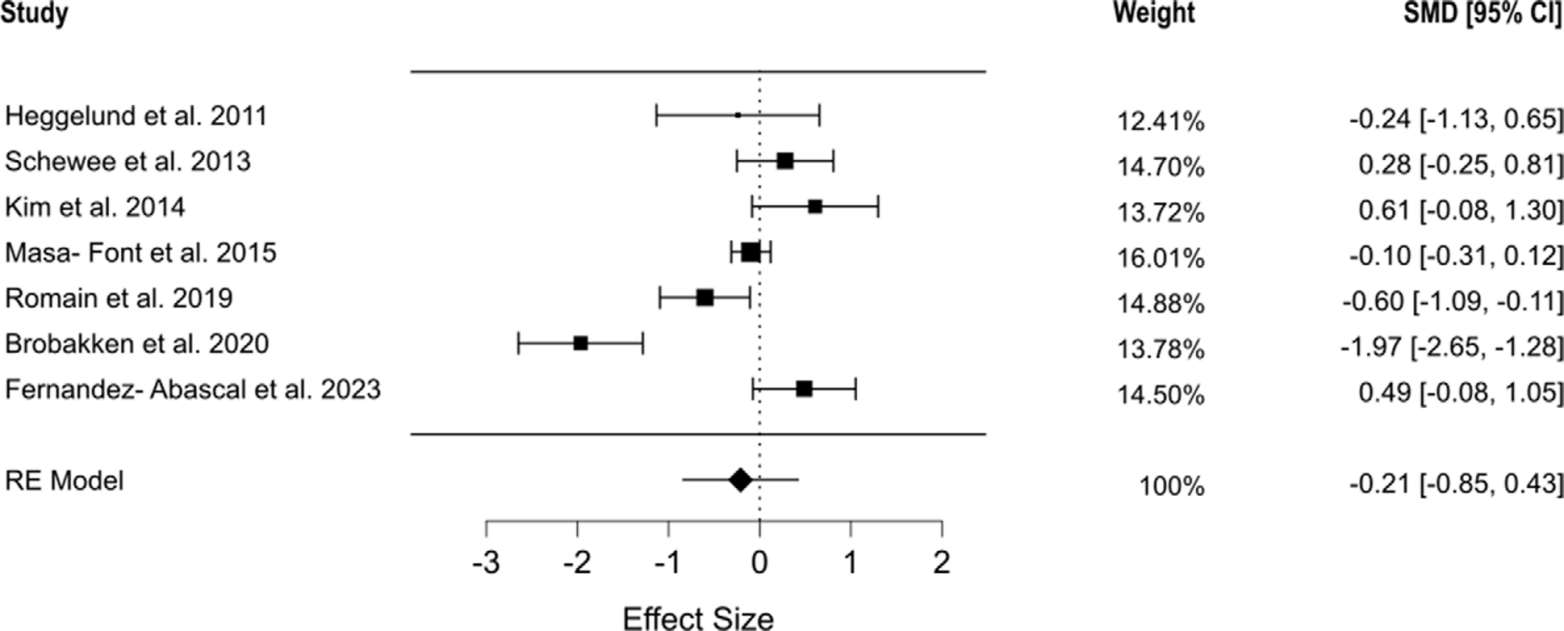
Forest plot showing the effect of physical exercise on diastolic blood pressure.

Seven studies examined systolic and diastolic blood pressure [43, 44, 46–49, 51]. For systolic blood pressure, the pooled data indicated no statistically significant effect of structured physical exercise interventions (SMD = 0.194, 95% CI [−0.115, 0.504], *p* = 0.219), with moderate heterogeneity observed (*Q* = 13.886, *I*^2^ = 61.258%, *p* = 0.031) (Figure 4). Similarly, diastolic blood pressure showed no significant changes (SMD = −0.21, 95% CI [−0.854, 0.434], *p* = 0.522), though the heterogeneity was substantial (*Q* = 42.724, *I*^2^ = 91.127%, *p* < 0.001) (Figure 5). The selection models showed no bias for either systolic (*p* = 0.294) or diastolic blood pressure (*p* = 0.31). These results were further validated by Egger’s regression tests (systolic: *p* = 0.589, diastolic: *p* = 0.773) [39, 52]. Detailed mean model estimates are provided in Supplementary Figure S2 for systolic blood pressure and Supplementary Figure S3 for diastolic blood pressure.

### HDL cholesterol

**Figure 6.**
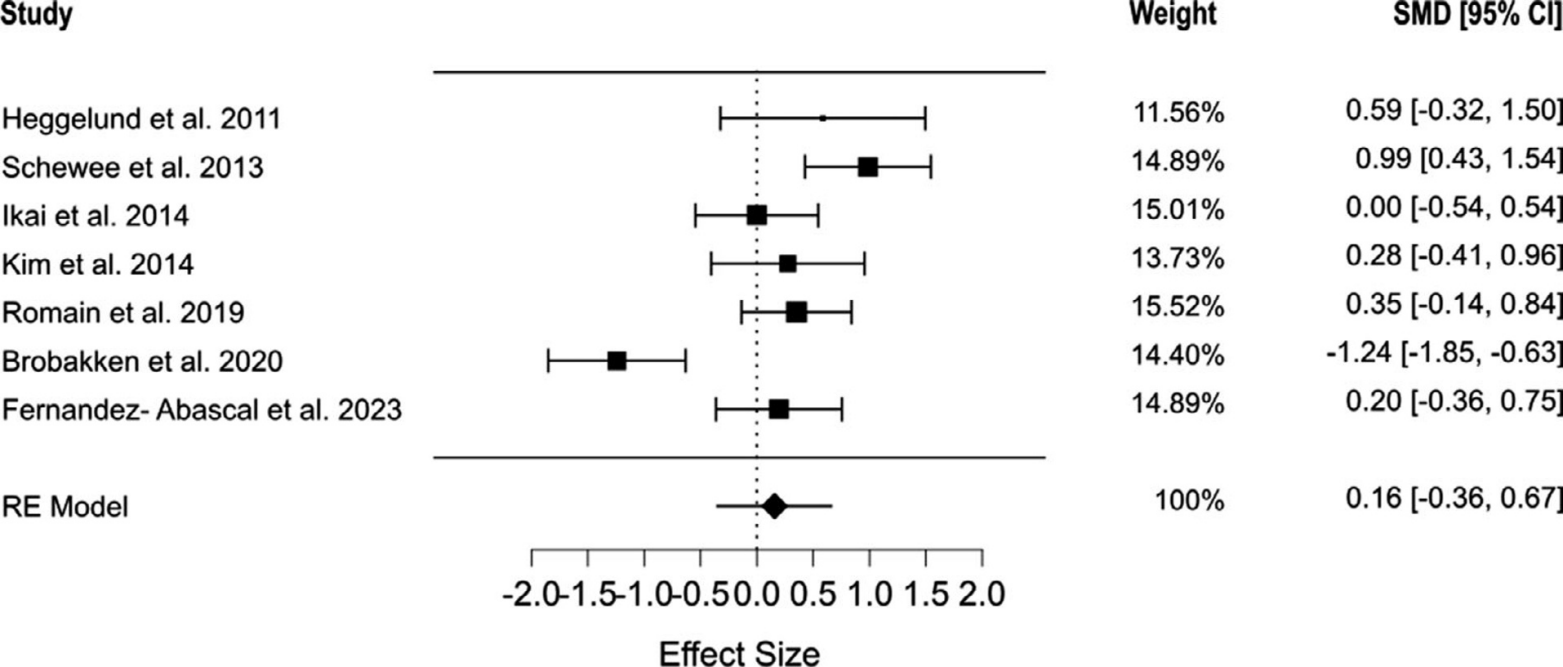
Forest plot of the effect of physical exercise on HDL cholesterol levels.

Seven studies evaluated the effects of exercise on high-density lipoprotein (HDL) cholesterol levels. Our findings revealed that exercise interventions did not significantly affect HDL cholesterol levels (SMD = 0.157, 95% CI [−0.36, 0.674], *p* = 0.551). However, substantial heterogeneity was observed across the included studies (Q = 30.617, *I*^2^ = 80.684%, *p* < 0.001) (Figure 6). No evidence of publication bias was detected (*p* = 0.421). This finding was further validated through Egger’s regression, confirming the absence of publication bias (*p* = 0.824) [39, 52]. The detailed estimates of the mean model are shown in Supplementary Figure S4.

### Triglycerides

**Figure 7.**
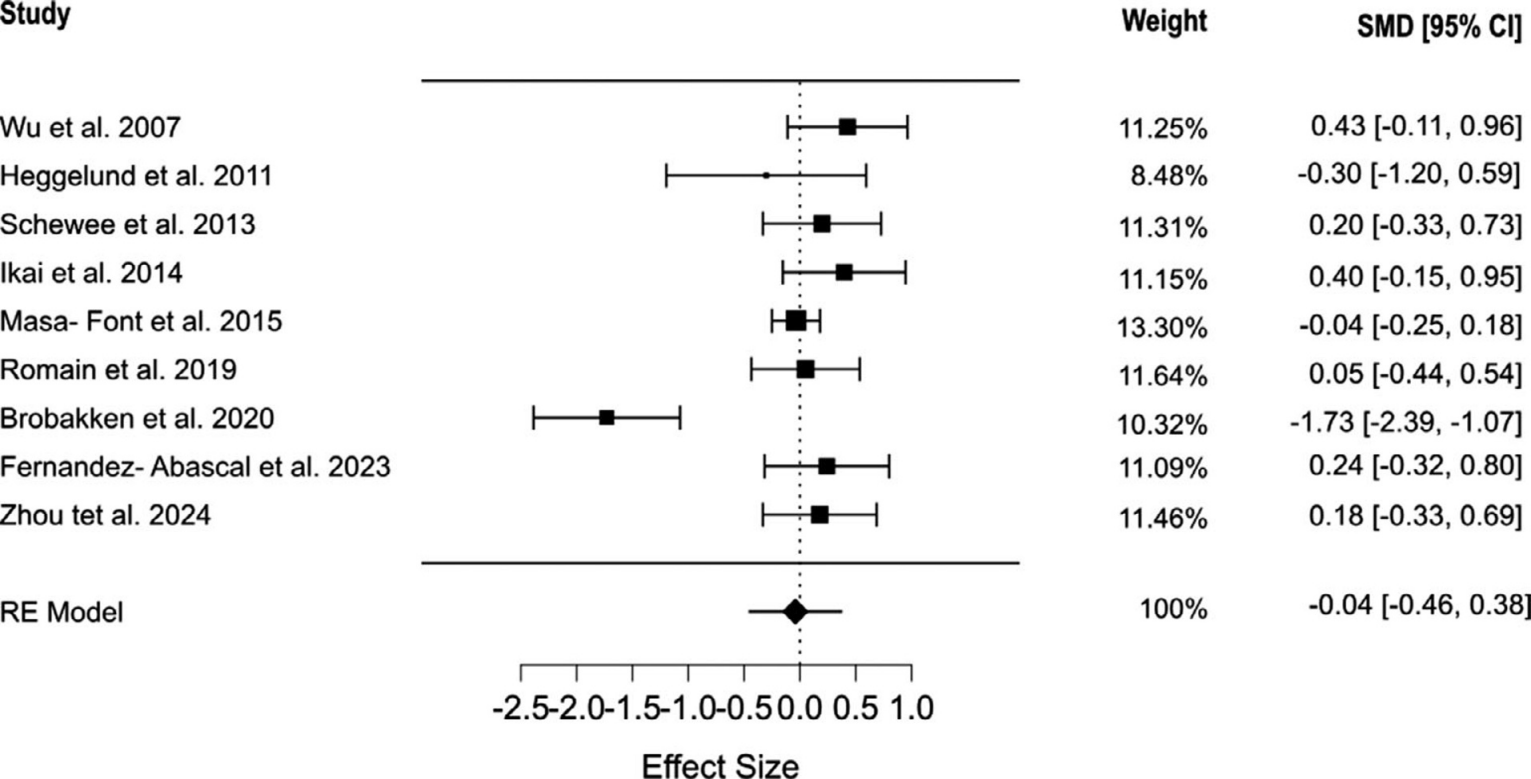
Forest plot showing the effect of physical exercise on triglyceride levels.

Nine studies assessed triglyceride levels [42–46, 48–51]. Our results indicated that physical exercise interventions did not significantly affect triglyceride levels (SMD = -0.041, 95% CI [−0.461, 0.38], *p* = 0.849). The included studies showed significant heterogeneity (*Q* = 33.432, *I*^2^ = 94.865%, *p* < 0.001) (Figure 7). The selection models reported no evidence of publication bias (*p* = 0.871), which was in line with the results of Egger’s regression (*p* = 0.413). The mean model estimates are presented in Supplementary Figure S5.

### Glucose

**Figure 8.**
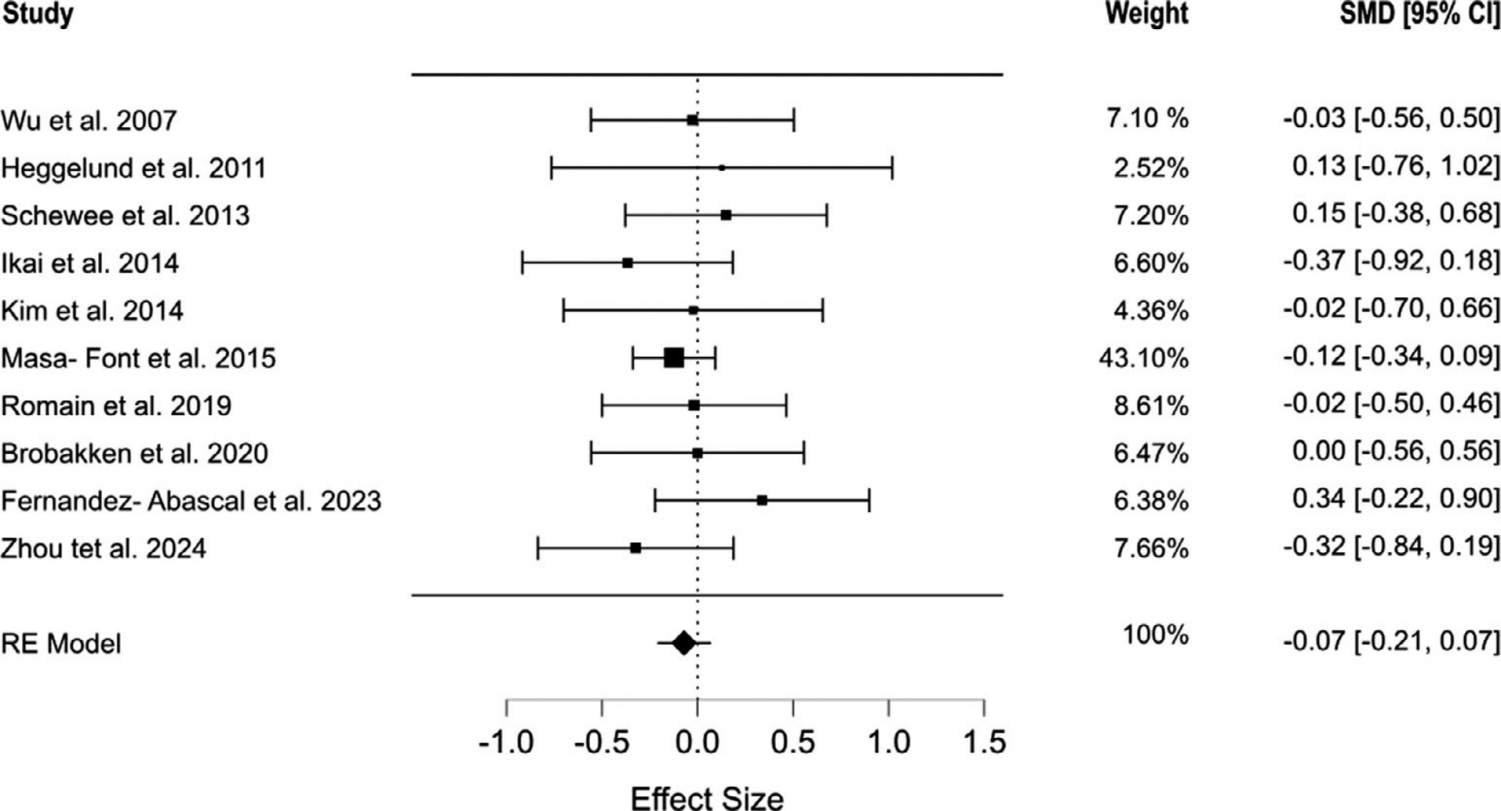
Forest plot showing the effect of physical exercise on glucose levels.

Ten studies evaluated the glucose levels [42–51]. Exercise intervention had no significant effect on glucose levels (SMD = −0.071, 95% CI [−0.213, 0.071], *p* = 0.326). No heterogeneity was observed among the included studies (*Q* = 5.323, *I*^2^ = 0%, *p* = 0.805) (Figure 8). The selection model analysis revealed no evidence of publication bias (*p* = 0.244). Egger’s regression supported this finding (*p* = 0.454) [39, 52]. The mean model estimates are presented in Supplementary Figure S6.

### Exercise interactions with medication

No studies have examined the possible interactions between physical exercise and medication (e.g., antipsychotics, antidepressants, or anxiolytics). Therefore, the secondary aim of this meta-analysis could not be addressed.

## Discussion

This meta-analysis evaluated whether structured physical exercise can improve metabolic syndrome components in individuals with psychotic disorders. Contrary to our hypothesis, we observed no significant benefits of exercise on metabolic syndrome. However, differences across studies make it difficult to draw definitive conclusions. Variability in exercise programs, how outcomes were measured, and the characteristics of study participants underscored the complexity of addressing metabolic dysregulation in this high-risk group. Critically, none of the included studies explored the interaction between exercise and psychotropic medications, which is a key factor that could influence the results. This gap prevented us from evaluating secondary objectives.

According to GRADE assessments, the overall certainty of evidence supporting the effects of physical exercise on metabolic outcomes in individuals with psychotic disorders was rated as low to moderate. This was mainly attributed to the presence of moderate-to-high heterogeneity across trials, risk of bias in several domains, and imprecision of the pooled estimates.

### Heterogeneity in exercise interventions and metabolic outcomes

Patients with severe mental illnesses, especially those with psychotic disorders such as schizophrenia, face an elevated risk of developing metabolic syndrome due to intertwined biological, inflammatory, and iatrogenic factors [7, 17, 18, 53]. Antipsychotics promote central obesity and insulin resistance through weight gain and adipocyte dysfunction [17, 18], while psychotic disorders related inflammation independently worsens cardiometabolic risk [9, 10, 20].

Despite the well-established link between metabolic syndrome and cardiovascular disease [12], the included trials assessed its components inconsistently (e.g., prioritizing isolated markers such as waist circumference or triglycerides over syndromic criteria). This methodological inconsistency underscores the need for standardized protocols in future studies [42–51].

Methodological heterogeneity in defining and measuring metabolic syndrome components significantly hampered cross-study comparability. Key examples include waist circumference measurement, protocols between the WHO-standardized midpoint (iliac crest to lower rib margin), umbilical positioning, and inconsistent subject posture or respiratory phase documentation. Despite universal reporting of triglyceride and HDL, lipid assessments were similarly compromised by variable fasting durations (8–12 h), sample processing methods, and assay techniques. This reflects nonadherence to the established diagnostic criteria (e.g., IDF and NCEP-ATP III). Future trials should implement standardized protocols (WHO STEPPS for anthropometry and CDC-NHLBI guidelines for lipids) with explicit methodological reporting. The development of harmonized COS for SMI metabolic research would enable meaningful evidence synthesis.

Notably, multimodal interventions combining aerobic and resistance training demonstrated modest benefits [46, 47], supporting that varied exercise regimens enhance metabolic flexibility [27, 29, 54]. On the other hand, trials using only aerobic or low-intensity workouts reported no benefits, suggesting that both exercise type and intensity play a critical role [48, 50, 51]. While high-intensity interval training (HIIT) has potential advantages, it is difficult to implement in this population owing to practical challenges, including motivation and tolerance issues. A key limitation of this study is the paucity of trials comparing exercise modalities. We could not determine the optimal exercise type for metabolic improvement with only two HIIT studies, one yoga trial, and two combined training regimens. This heterogeneity prevents robust recommendations regarding specific protocols (e.g., HIIT vs. resistance training) and underscores the need for head-to-head comparative trials.

We aggregated data across schizophrenia, schizoaffective disorder, and schizophreniform disorder owing to their shared cardiometabolic risk profiles and universal exposure to second-generation antipsychotics (SGAs), which frequently induce weight gain, dyslipidemia, and glucose intolerance. While the illness course and symptomatology show some heterogeneity, this grouping aligns with established psychiatric research paradigms and reflects clinical practice realities. Future studies with larger sample sizes may facilitate diagnostic subgroup analysis.

### Blood pressure and exercise intensity

Exercise interventions were not associated with significant reductions in systolic or diastolic blood pressure compared to nonexercise controls, with moderate to high heterogeneity across studies. These findings are consistent with prior evidence suggesting that exercise alone, without adjunctive dietary or pharmacological strategies, may be insufficient to treat hypertension in individuals with psychotic disorders [7]. Notably, antipsychotic medications have been associated with hypertension, especially second-generation medications such as clozapine and olanzapine, through mechanisms such as weight gain, sodium retention, and sympathetic activation [17, 18, 55]. While Bredin et al. [56] reported blood pressure improvements after 12 weeks of aerobic or resistance training, their cohort lacked severe psychiatric comorbidities, potentially inflating generalizability. Exercise intensity is a major moderator of training outcomes; studies involving high-intensity interval training (HIIT; 70–95% HR peak) or vigorous aerobic protocols (50–90% HR_max_) [43, 44] demonstrated greater metabolic adaptations than moderate-intensity regimens [48, 49], in line with the dose–response relationships observed in nonpsychiatric populations [57–59]. However, HIIT may not be feasible in psychosis, where motivational deficits, sedation with antipsychotics, and cognitive impairments pose significant barriers. Large trials stratifying patients according to illness severity and medication regimens are needed to clarify optimal intensity thresholds.

### Lipid profiles and medication interactions

Despite the role of aerobic exercise in improving lipid metabolism [33], our meta-analysis did not find any significant effects on triglyceride or HDL cholesterol levels. This discrepancy likely reflects pronounced metabolic dysregulation in the SMI. Antipsychotics directly exacerbate lipid abnormalities via hepatic lipogenesis (endogenous hepatic synthesis of fatty acids from carbohydrates) and adipocyte dysfunction (impaired function of fat-storing cells) [14, 60]. For instance, clozapine and olanzapine elevate triglyceride levels by up to 40% and reduce HDL levels by 15–20% in psychotic disorders [17, 18, 55], which may overwhelm the modest lipid-modulating capacity of exercise. A pilot study demonstrating HDL reduction at week 12 of training [56] further highlights the complexity of the lipid response in medicated populations. Although exercise may mitigate antipsychotic-induced hypertriglyceridemia without compromising psychiatric stability [61], the mechanisms involved remain unclear. The proposed pathways include enhanced lipoprotein lipase activity and β oxidation [62], though inflammation and oxidative stress, which are hallmarks of psychosis, may blunt these adaptations. Future studies must incorporate direct biomarkers (e.g., apolipoprotein B and LDL particle size) and control for medication dose, duration, and polypharmacy to disentangle these interactions.

Standardization extends beyond measurement techniques to encompass the diagnostic criteria. Only 3 of 10 included studies applied established metabolic syndrome definitions (e.g., IDF or NCEP-ATP III), while others reported isolated components [44, 46, 48]. These risks misclassify outcomes and dilute actual intervention effects. Future work should mandate adherence to internationally recognized criteria and report individual components along with composite syndrome status.

### Glucose metabolism and adherence challenges

Data from all studies in this meta-analysis assessed glucose levels after exercise interventions, but limited improvements were observed despite exercise is established to enhance insulin sensitivity [3, 10, 63]. Previous studies using an intervention based on aerobic training alone [48, 50] did not improve hyperglycemia, perhaps because of suboptimal intensity, insufficient duration, or poor adherence. Of note, the only adherence report – a critical determinant of exercise efficacy – reflected data at point, FFD 30 months and FFD 40 months in five trials with an adherence range of 60–75% [44, 48, 49]. However, one trial reported a 50% dropout rate in the intervention arm, which was primarily attributed to motivational deficits and program duration [44].

Attrition of this type is consistent with larger evidence associating poor adherence in SMI populations with psychosocial obstacles (e.g., cognitive impairment and social isolation) and adverse consequences of medications (e.g., sedation and fatigue) [22]. The lack of standardized adherence metrics across studies – half omitted attendance thresholds or compliance criteria – further complicates the interpretation. These findings underscore the necessity of integrating behavioral strategies (e.g., motivational interviewing and peer support) to sustain engagement in this population.

The lack of glucose-lowering effects may also indicate profound metabolic dysregulation inherent to psychotic disorders. Antipsychotics, including olanzapine and clozapine, modulate insulin signaling via direct β-cell toxicity and adipose tissue inflammation [64, 65], which could cancel out exercise-induced benefits. Furthermore, exercise prescriptions in the included trials rarely accounted for medication specific metabolic risks or individualized glycemic targets. Future interventions should prioritize stratified designs that control for antipsychotic class, dose, and illness chronicity while incorporating continuous glucose monitoring to capture dynamic responses.

### Antipsychotic medications: A critical confounder

All participants were prescribed antipsychotics, which are known to promote weight gain, dyslipidemia, and insulin resistance [64, 66, 67]. One trial exclusively included patients treated with clozapine [20, 50], a subgroup with a greater metabolic risk [64, 65]. This timing is further challenged by the progressive metabolic burden in chronic (vs. first-episode) psychosis [65, 66, 68, 69].

Although structured exercise interventions offer multiple health benefits, this meta-analysis found no evidence that exercise improves metabolic syndrome in individuals with SMI receiving antipsychotic treatment. Therefore, although physical activity should remain a safe and broadly beneficial component, it appears insufficient to overcome the metabolic burden associated with antipsychotics. Pharmacodynamic interactions, such as how exercise alters drug metabolism or receptor sensitivity, are understudied, but should be urgently investigated [69].

In all trials selected for inclusion, participants were administered antipsychotics [42–51], known independent agents of metabolic dysregulation via mechanisms of weight gain, hypertriglyceridemia, and insulin resistance [17, 18, 64]. Reversible with dose reduction, second-generation antipsychotics (SGAs) such as clozapine, olanzapine, risperidone, and quetiapine worsen cardiovascular risk through direct actions on lipid metabolism (elevating triglycerides, reducing HDL) and glucose homeostasis [63, 70, 71]. Clozapine, in particular, demonstrates the most severe metabolic liabilities, with studies reporting a fourfold increased risk of type 2 diabetes and 20–25% weight gain within the first year of treatment [64, 65].

Critically, none of the trials examined exercise–psychotropic medication interactions, despite the possibility that physical activity may modulate drug pharmacokinetics (e.g., hepatic CYP450 enzyme activity) or counteract receptor-mediated metabolic harm (e.g., 5-HT2C antagonism-induced hyperphagia) [72, 73]. This limitation greatly hinders our ability to separate the independence of exercise as a therapeutic modality from its potential ameliorative effect as a treatment adjunct (e.g., metabolic salvage therapy) in drug-treated populations.

### Chronicity and the case for early intervention

The higher proportion of cases classified as chronic psychosis in our analysis [42–51] is consistent with the progressive nature of metabolic syndrome in SMI, whereby long exposure to antipsychotic medications and illness-related determinants (e.g., chronic inflammation, sedentariness) contribute to increased risk over time [65, 66, 68]. In contrast, patients with first-episode disease have a lower metabolic burden of morbidity, leaving a crucial time window for early intervention [68, 74]. Structured exercise may be protective when significant weight gain and insulin resistance begin, and exercise prevention studies initiated upon illness onset may help counter eating-induced weight gain and insulin resistance before irreversible pathophysiological derangements occur [74]. However, pragmatic barriers, including motivational deficits, medication induced fatigue, and limited access to tailored programs, hinder adherence in this population [75]. Adding exercise-coordinated specialty care models, nutritional counseling, and peer support may enhance engagement and sustainability. Our findings must be interpreted in the light of several limitations. The small, heterogeneous sample size of trials and inconsistent outcome report constrain generalizability. Although statistical tests did not detect publication bias, this remains plausible, given the niche focus of metabolic interventions in psychosis [42–51]. Critically, intervention heterogeneity, marked by disparate exercise modalities, intensities, and durations, precluded dose–response insights, while the pervasive metabolic effects of second-generation antipsychotics (SGAs) likely obscure exercise-specific benefits.

Furthermore, adherence variability, compounded by poor reporting and high attrition rates, introduces potential bias. To address these gaps, future studies should standardize metabolic syndrome criteria (e.g., incorporating direct biomarkers such as insulin sensitivity), stratified analyses by antipsychotic class, dose, and illness chronicity, and integrate evidence-based behavioral strategies to enhance adherence. Such refinements are essential for isolating the therapeutic potential of exercise within the complex biopsychosocial landscape of severe mental illnesses.

Future studies should explicitly evaluate the interactions between specific antipsychotic agents and exercise-induced metabolic responses. This includes stratifying participants by antipsychotic type (e.g., clozapine vs. aripiprazole) and dose and exploring potential mechanisms such as altered receptor binding, hepatic metabolism (e.g., CYP450 modulation), and downstream effects on lipid and glucose regulation. A deeper understanding of these interactions will enable more tailored and clinically relevant exercise prescriptions for this population.

## Conclusions

While structured exercise alone yields disappointing metabolic outcomes in chronic psychosis, targeted interventions are promising for precise clinical scenarios. Early intervention cohorts, particularly first-episode patients before irreversible metabolic derangement, and those on lower risk antipsychotics (e.g., aripiprazole/lurasidone rather than olanzapine/clozapine) may derive robust benefits from multimodal regimens combining aerobic and resistance training ≥3x/week at 60–80% HR_max_. Consequently, we advocate integrating exercise into a broader cardiometabolic risk mitigation strategy: (1) optimizing pharmacotherapy to minimize obesogenic agents, (2) codelivering structured dietary interventions, and (3) employing metformin for antipsychotic-induced hyperglycemia, with continuous metabolic monitoring. Future studies must stratify by illness chronicity, medication risk profiles, and exercise modalities to identify responders.

Future trials should prioritize stratification by antipsychotic type and dosage and rigorously investigate medication specific interactions with exercise. Such approaches are essential to improve the precision of clinical recommendations and develop effective, individualized interventions that mitigate cardiometabolic risk in individuals with psychotic disorders.

## Supporting information

10.1192/j.eurpsy.2025.10064.sm001 Ancín-Osés et al. supplementary material 1 Ancín-Osés et al. supplementary material

10.1192/j.eurpsy.2025.10064.sm002 Ancín-Osés et al. supplementary material 2 Ancín-Osés et al. supplementary material

10.1192/j.eurpsy.2025.10064.sm003 Ancín-Osés et al. supplementary material 3 Ancín-Osés et al. supplementary material

## Data Availability

Data available from the authors upon reasonable request.

## Supplementary material

The supplementary material for this article can be found at http://doi.org/10.1192/j.eurpsy.2025.10064.
