# Supplementary material for: Effects of physical exercise on metabolic syndrome in psychotic disorders: A systematic review with meta-analysis of randomized controlled trials
Source: Eur Psychiatry. 2025 Jul 18;68(1):e101. doi: 10.1192/j.eurpsy.2025.10064 (PMC12344471; doi:10.1192/j.eurpsy.2025.10064)
Supplement: Ancín-Osés et al. supplementary material 3 — Ancín-Osés et al. supplementary material [file S0924933825100643sup003.docx]

Search strategy implemented in four electronic databases and results of total records.

| **Search strategy implemented across for electronic databases and results of total records retrieved on 10 October 2024** | | |
| --- | --- | --- |
| **PUBMED** | (“schizophrenia”[MeSH] OR "schizophrenia, catatonic"[Mesh] OR "schizophrenia, disorganized"[Mesh] OR "schizophrenia, paranoid"[Mesh] OR “Schizoaffective disorder” [title/abstract] OR “schizophreniform disorder” [title/abstract] OR “Psychotic Disorders”[Mesh] OR "schizophren*"[title/abstract] OR “psychosis” [title/abstract] OR “psychoses” [title/abstract] OR “first episode psychosis” [title/abstract] OR “delusional disorder”[title/abstract]) AND ("Exercise Therapy"[Mesh] OR "Exercise Movement Techniques"[Mesh] OR "Resistance Training"[Mesh] OR "Muscle Stretching Exercises"[Mesh] OR "Exercise"[Mesh] OR Exercise Isometric [title/abstract] OR Exercise Aerobic [title/abstract] OR Aerobic Exercises [title/abstract] OR Aerobic Exercise [title/abstract] OR Training Resistance [title/abstract] OR Strength Training [title/abstract] OR Weight Lifting [title/abstract] OR Strengthening Program [title/abstract] OR Weight Bearing [title/abstract] OR Exercises [title/abstract] OR Physical Exercise [title/abstract] OR Physical Exercises [title/abstract] OR Isometric Exercises [title/abstract] OR Isometric Exercise [title/abstract] OR Aerobic Exercises [title/abstract] OR Aerobic Exercise [title/abstract] OR Exercise Therapies [title/abstract] OR Strength Training [title/abstract] OR Strengthening Programs [title/abstract] OR Weight Lifting Exercise Program [title/abstract] OR Weight Bearing Strengthening Program [title/abstract] OR Weight Bearing Exercise Program [title/abstract]) AND (randomized Controlled Trial [title/abstract] OR Controlled Clinical Trial [title/abstract] OR Randomized Controlled Trials [title/abstract] OR Random* [title/abstract] OR Double-Blind [title/abstract] OR Single-Blind [title/abstract] OR Clinical Trials [title/abstract]) | 575 |
| **WOS** | (AB=(“schizophrenia” OR “catatonic schizophrenia” OR " disorganised schizophrenia” OR “paranoid schizophrenia” OR “Schizoaffective disorder” OR “schizophreniform disorder” OR “psychotic disorders” OR "schizophren*" OR “psychosis” OR “psychoses” OR “first episode psychosis” OR “delusional disorder”)) AND (TS=("Exercise Therapy" OR "Exercise Movement Techniques" OR "Resistance Training" OR "Muscle Stretching Exercises" OR "Exercise" OR Exercise Isometric OR Exercise Aerobic OR Aerobic Exercises OR Aerobic Exercise OR Training Resistance OR Strength Training OR Weight Lifting OR Strengthening Program OR Weight Bearing OR Exercises OR Physical Exercise OR Physical Exercises OR Isometric Exercises OR Isometric Exercise OR Aerobic Exercises OR Aerobic Exercise OR Exercise Therapies OR Strength Training OR Strengthening Programs OR Weight Lifting Exercise Program OR Weight Bearing Strengthening Program OR Weight Bearing Exercise Program)) AND (TS=(randomized Controlled Trial OR Controlled Clinical Trial OR Randomized Controlled Trials OR Random* OR Double-Blind OR Single-Blind OR Clinical)) | 1726 |
| **CINAHL** | S1: AB “schizophrenia” OR AB "catatonic schizophrenia” OR AB "disorganised schizophrenia” OR AB "paranoid schizophrenia" OR AB “schizoaffective disorder” OR AB “schizophreniform disorder” OR AB "psychotic disorders" OR AB "schizophren*" OR AB "psychosis" OR AB "psychoses" OR AB “first episode psychosis" OR AB “delusional disorder”  S2: TX "Exercise Therapy" OR TX "Exercise Movement Techniques" OR TX "Resistance Training" OR TX "Muscle Stretching Exercises" OR TX "Exercise" OR TX Exercise Isometric OR TX Exercise Aerobic OR TX Aerobic Exercises OR TX Aerobic Exercise OR TX Training Resistance OR TX Strength Training OR TX Weight Lifting OR TX Strengthening Program OR TX Weight Bearing OR TX Exercises OR TX Physical Exercise OR TX Physical Exercises OR TX Isometric Exercises OR TX Isometric Exercise OR TX Aerobic Exercises OR TX Aerobic Exercise OR TX Exercise Therapies OR TX Strength Training OR TX Strengthening Programs OR TX Weight Lifting Exercise Program OR TX Weight Bearing Strengthening Program OR TX Weight Bearing Exercise Program  S3: TX randomized Controlled Trial OR TX Controlled Clinical Trial OR TX Randomized Controlled Trials OR TX Random* OR TX Double-Blind OR TX Single-Blind OR TX Clinical  S1 AND S2 AND S3 | 910 |
| **APA PsycINFO** | S1: AB “schizophrenia” OR AB "catatonic schizophrenia” OR AB "disorganised schizophrenia” OR AB "paranoid schizophrenia" OR AB “schizoaffective disorder” OR AB “schizophreniform disorder” OR AB "psychotic disorders" OR AB "schizophren*" OR AB "psychosis" OR AB "psychoses" OR AB “first episode psychosis" OR AB “delusional disorder”  S2: TX "Exercise Therapy" OR TX "Exercise Movement Techniques" OR TX "Resistance Training" OR TX "Muscle Stretching Exercises" OR TX "Exercise" OR TX Exercise Isometric OR TX Exercise Aerobic OR TX Aerobic Exercises OR TX Aerobic Exercise OR TX Training Resistance OR TX Strength Training OR TX Weight Lifting OR TX Strengthening Program OR TX Weight Bearing OR TX Exercises OR TX Physical Exercise OR TX Physical Exercises OR TX Isometric Exercises OR TX Isometric Exercise OR TX Aerobic Exercises OR TX Aerobic Exercise OR TX Exercise Therapies OR TX Strength Training OR TX Strengthening Programs OR TX Weight Lifting Exercise Program OR TX Weight Bearing Strengthening Program OR TX Weight Bearing Exercise Program  S3: TX randomized Controlled Trial OR TX Controlled Clinical Trial OR TX Randomized Controlled Trials OR TX Random* OR TX Double-Blind OR TX Single-Blind OR TX Clinical  S1 AND S2 AND S3 | 693 |
| **Overall** | | **3904** |


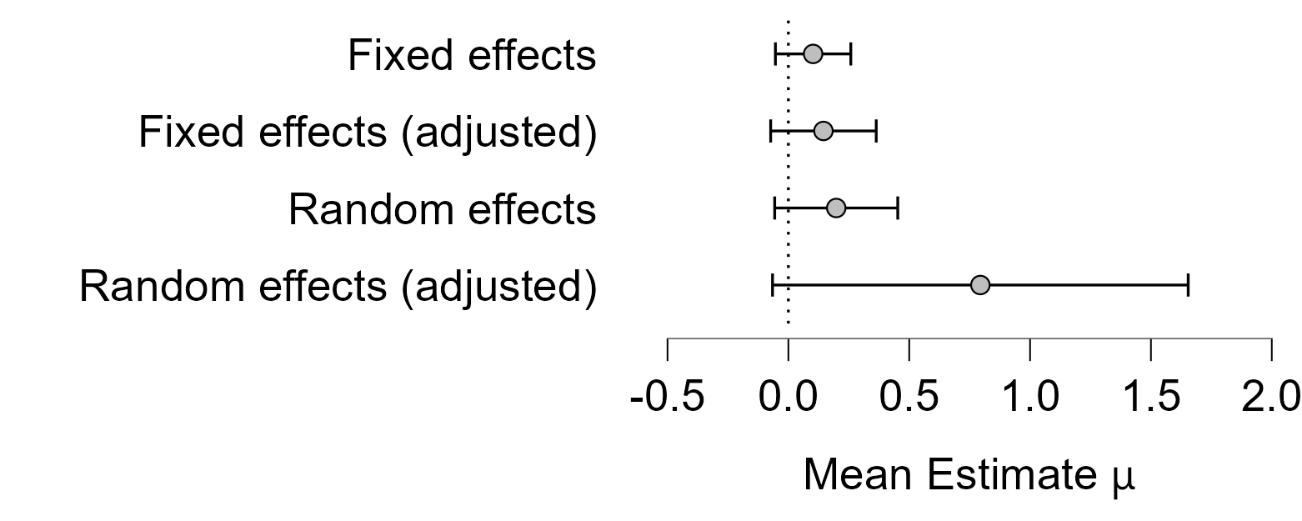


**Supplementary Figure 1.** **Mean model estimations of RCTs assessing waist circumference.**

**
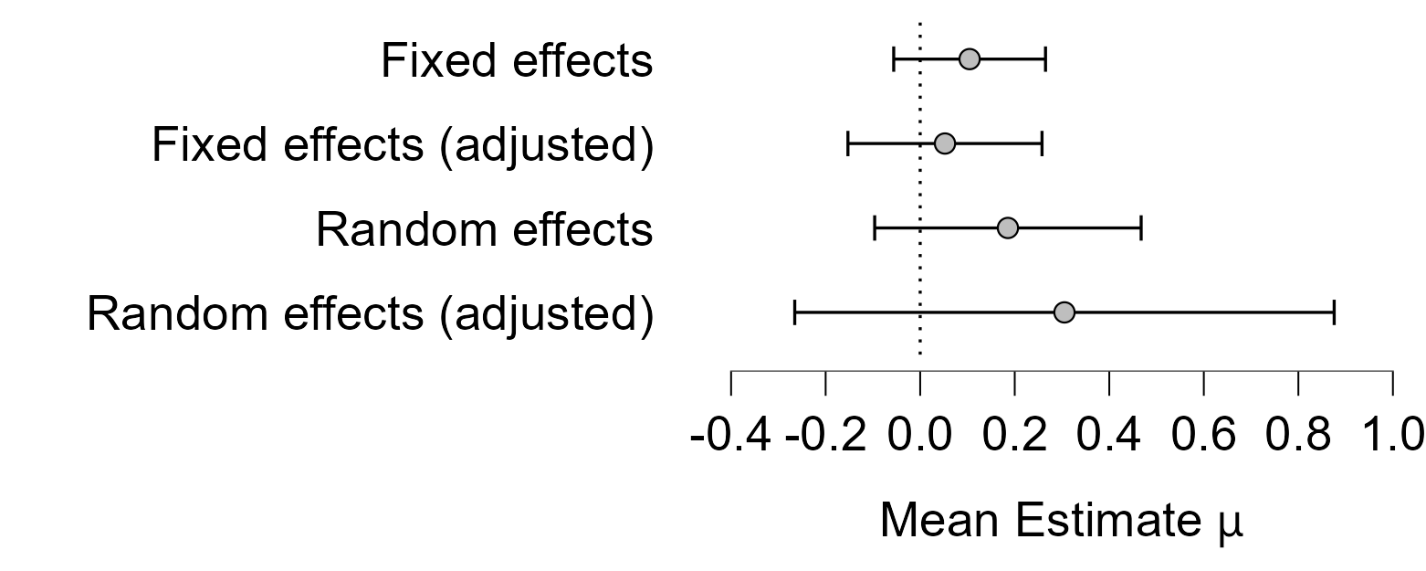
**

**Supplementary Figure 2.** **Mean model estimations of RCTs assessing systolic blood pressure.**

**
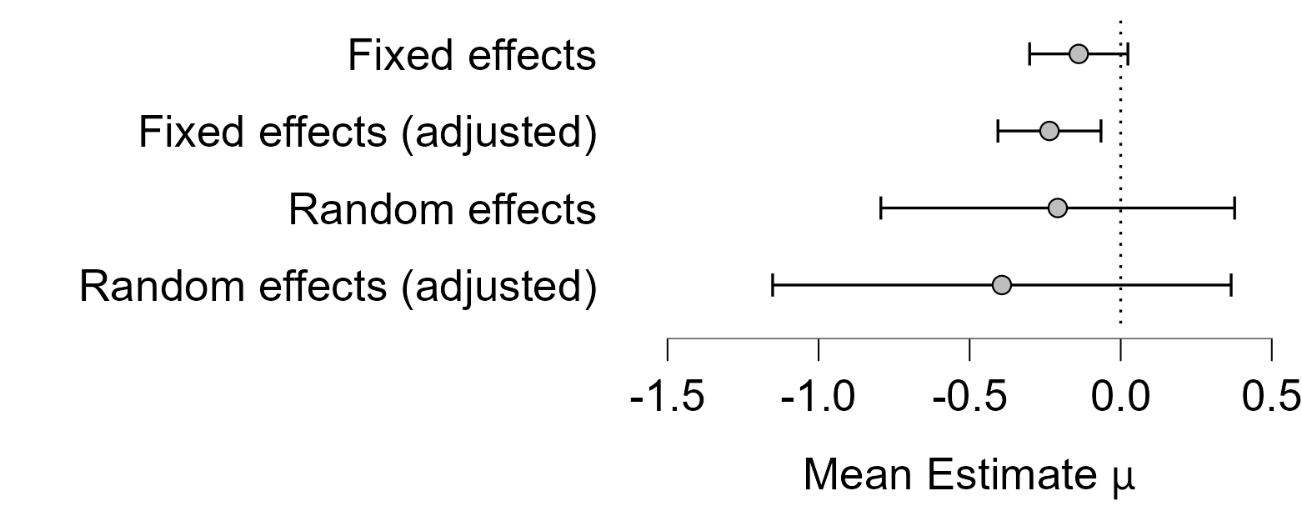
**

**Supplementary Figure 3.** **Mean model estimations of RCTs assessing diastolic blood pressure.**

**
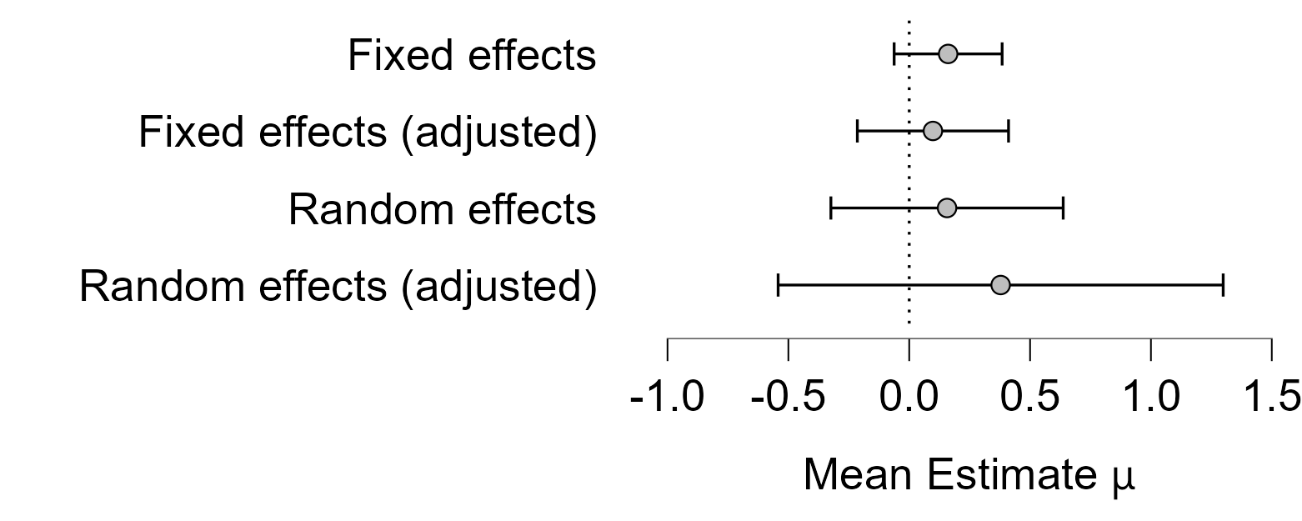
**

**Supplementary Figure 4.** **Mean model estimations of RCTs assessing HDL cholesterol.**

**
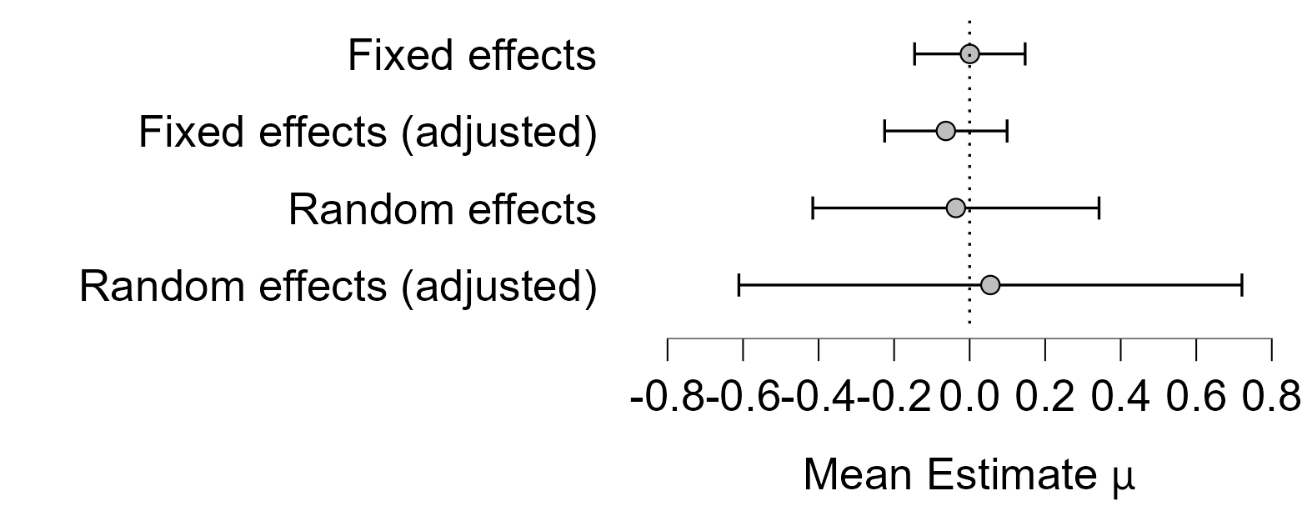
**

**Supplementary Figure 5.** **Mean model estimations of RCTs assessing triglycerides.**

**
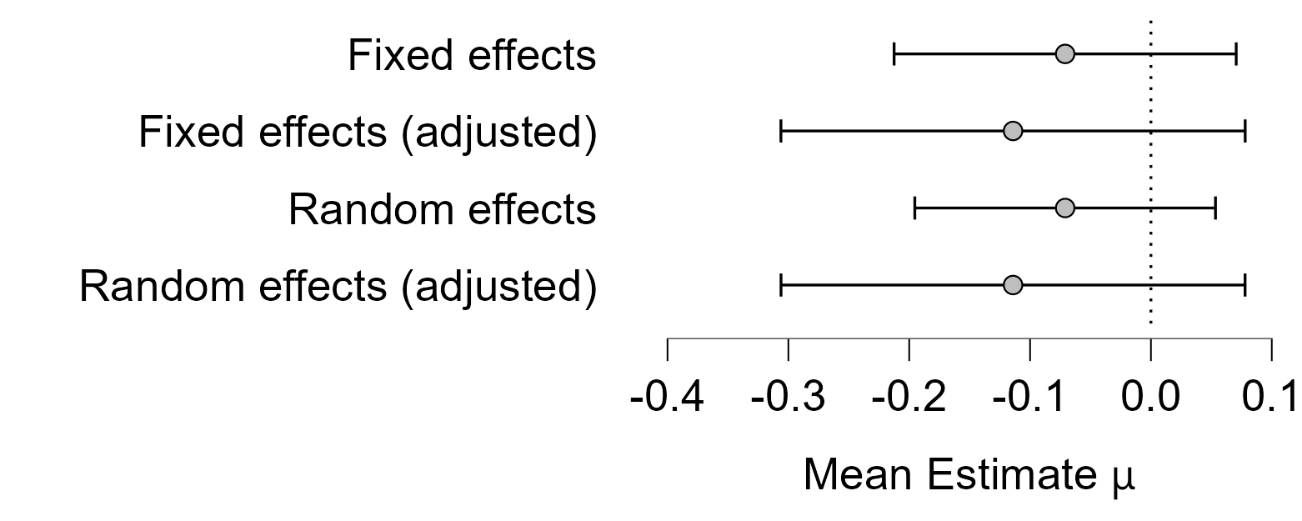
**

**Supplementary Figure 6.** **Mean model estimations of RCTs assessing glucose levels.**
